# The Mortality Risk of Proton Pump Inhibitors in 1.9 Million US Seniors: An Extended Cox Survival Analysis

**DOI:** 10.1016/j.cgh.2021.01.014

**Published:** 2021-01-13

**Authors:** Seo H. Baik, Kin-Wah Fung, Clement J. McDonald

**Affiliations:** Lister Hill National Center for Biomedical Communications, National Library of Medicine, US National Institutes of Health, Bethesda, Maryland

**Keywords:** Proton Pump Inhibitors, All-Cause Mortality, Protopathic Bias, Time-Varying Propensity Scores, Extended Cox Regression

## Abstract

**BACKGROUND & AIMS:**

Observational studies have linked proton pump inhibitors (PPIs) with increased risk of mortality and other safety outcomes, in contradiction with a recent PPI randomized controlled trial (RCT). Observational studies may be prone to reverse causality, where deaths are attributed to the treatment rather than the conditions that are treated (protopathic bias).

**METHODS:**

We analyzed an incident drug user cohort of 1,930,728 elderly Medicare fee-for-service beneficiaries to evaluate the PPI-associated risk of death with a Cox regression analysis with time-varying covariates and propensity score adjustments. To correct for protopathic bias which occurs when a given drug is associated with prodromal signs of death, we implemented a lag-time approach by which any study drug taken during a 90-day look-back window before each death was disregarded.

**RESULTS:**

Among 1,930,728 study individuals, 80,972 (4.2%) died during a median 3.8 years of follow-up, yielding an overall unadjusted death rate/1000 person-years of 9.85; 14.31 for PPI users and 7.93 for non- users. With no lag-time, PPI use (vs no use) was associated with 10% increased mortality risk (adjusted HR[1.10; 95% CI 1.08–1.12). However, with a lag-time of 90 days, mortality risk associated with PPI use was near zero (adjusted HR[1.01; 95% CI 0.99–1.02).

**CONCLUSION:**

Given the usage patterns of PPIs in patients with conditions that may presage death, protopathic bias may explain the association of PPIs with increased risk of death reported in observational studies.

Proton pump inhibitors (PPIs) and their alternative histamine-2 receptor agonists (H2RAs) are indicated for treatment of heartburn and gastroesophageal reflux disease.^1^ Hospitalized patients are often given either PPIs or H2RAs to prevent stress ulcers, and PPIs have been used to treat 80% of patients admitted to internal medicine services.^2^ In recent decades, both PPIs and H2RAs became available for purchase without a prescription,^3^ and the use of PPIs in particular has surged.^4^

Observational studies have suggested that PPI use increases the risk of pneumonia,^5^ *Clostridium difficile* infection,^6^ osteoporosis/fractures,^7^ renal failure,^8^ cardiac events,^9^ and death.^10^ Two large studies from the Department of Veterans Affairs (VA) reported 17%–25%^10,11^ increased mortality risk with PPIs compared with H2RAs. The media has publicized warnings from these studies about the use of PPIs.^12^

Contradicting these studies, a 17,598 patient, 3-year, randomized controlled trial (RCT) of a popular PPI reported no significant mortality risks or other safety outcomes.^13^ However, there was residual uncertainty. The confidence interval (CI) for all-cause mortality was wide enough (0.92–1.15) to allow the possibility of a small increase in mortality risk.

The Centers for Medicare and Medicaid Services’ Virtual Research Data Center is a very large database carrying prescription drug and inpatient/outpatient encounter data. It has more complete capture than VA databases for Medicare-eligible Veterans who receive a major share of their health care from outside the VA health system.^14^ In order to clarify the degree of risk from PPI use, we studied their effect on mortality in patients in the Virtual Research Data Center.

## Materials and Methods

### Study Population and Selection Criteria

We obtained all prescription and encounter claims data for a 20% random sample of Medicare Prescription Drug (Part D) coverage enrollees who first enrolled in Medicare near 65 years of age (779–781 months old), and from 2007 (the first full year of Part D claim availability) to 2017.

We limited our study population to fee-for-service enrollees, for whom Medicare Parts A and B claims would exist. Because 30% of Part D enrollment may lag Medicare enrollment by 60 days or longer, we followed them from their Part D enrollment, while accounting for left truncation,^15^ until they (1) died, (2) switched to a capitated plan, (3) disenrolled from Medicare, or (4) reached the end of our data availability on December 31, 2017, whichever came first. We only included patients who were in our study for more than 6 months to assure enough follow-up time, and those continuously enrolled in both Parts A and B during the follow-up to assure availability of medical claims. We washed out individuals with claims for PPIs or H2RAs in the first 6 months in Medicare Part D to obtain an incident cohort. Our primary analysis focused on this cohort but we report a few observations from the full cohort. To allow better comparison with results of the VA studies, we also created a special Medicare cohort that included only male beneficiaries and entered in 2007 or 2008 to provide a sex distribution and follow-up duration comparable to the VA population (see Supplementary Material A).

### Medication Data

We examined the effect of H2RAs and PPIs as separate classes. Our PPI class included all PPIs available in the United States (omeprazole, pantoprazole, esomeprazole, lansoprazole, dexlansoprazole, rabeprazole). Our H2RA class included all H2RAs available in the United States (ranitidine, famotidine, cimetidine, nizatidine). We did not take into account brand name and strength distinctions within drug class, and report usage trends by drug class and generic ingredient within class.

### Chronic Condition Data

We included 46 Medicare chronic conditions with >1% prevalence in our descriptive and Cox regression analyses. Medicare specifies the onset of each condition by algorithms and records the dates in the Master Beneficiary Summary File.^16^

For patients who took only PPIs, only H2RAs, both PPIs and H2RAs, or neither PPIs nor H2RAs, we separately report prevalence of death, unadjusted death rate, sociodemographics, and the prevalence of selected chronic conditions.

### Statistical Analysis

To explore the independent effect of PPIs and H2RAs on all-cause mortality, we employed an extended Cox regression with the number of months on study as time scale. Extended Cox regression analysis protects against the immortal time bias and violation of proportional hazards assumption.^17,18^ In our analysis, we included covariates for sex, race, rural residence indicator and the degree of low-income-subsidy (LIS) (a measure of economic status): (1) dual eligible, having income at or below 135% Federal Poverty Line (FPL); (2) nondual LIS, having income in 135–150% FPL; and (3) nondual no LIS, having income above 150% FPL.^19^ We included binary flags for each of the 46 Medicare chronic conditions (as proxies for medical burden). We included the calendar year of the subject’s Part D enrollment to adjust for secular trends. In order to mitigate selection bias toward the use of each study drug, we ran all regression analyses with propensity scores as additional adjustments (see Supplementary Material B).

We treated the use of PPIs, H2RAs, the degree of LIS, 46 chronic conditions, and the propensity scores as time-varying covariates. Sex, race, and rural residence were defined by their status at the time of Medicare enrollment and were the only time-fixed covariates. The disease covariates were chronic diseases, so we considered them as always present after their onset and absent before then. The values of other time-varying covariates were reset at the time of each death in the Cox regression. We used Efron’s adjustment for tied events.^20^

Protopathic bias (reverse causality) occurs when an intervention is associated with prodromal signs of that outcome.^21^ Observational studies on PPIs may be especially prone to protopathic bias because PPIs are prescribed for disorders and events such as intensive care unit (ICU) admissions, renal dialysis, and anticoagulant and antiplatelet treatment that can be prodromal to death.^22–25^ To protect against this bias, we used 90-day lag times and disregarded any study drug taken during a lookback window 90 days prior to each event (death) date. We conducted the analysis with and without time lags.

All authors had access to the study data and reviewed and approved the final manuscript.

## Results

### Secular Trends and Descriptive Statistics

**Figure 1.**
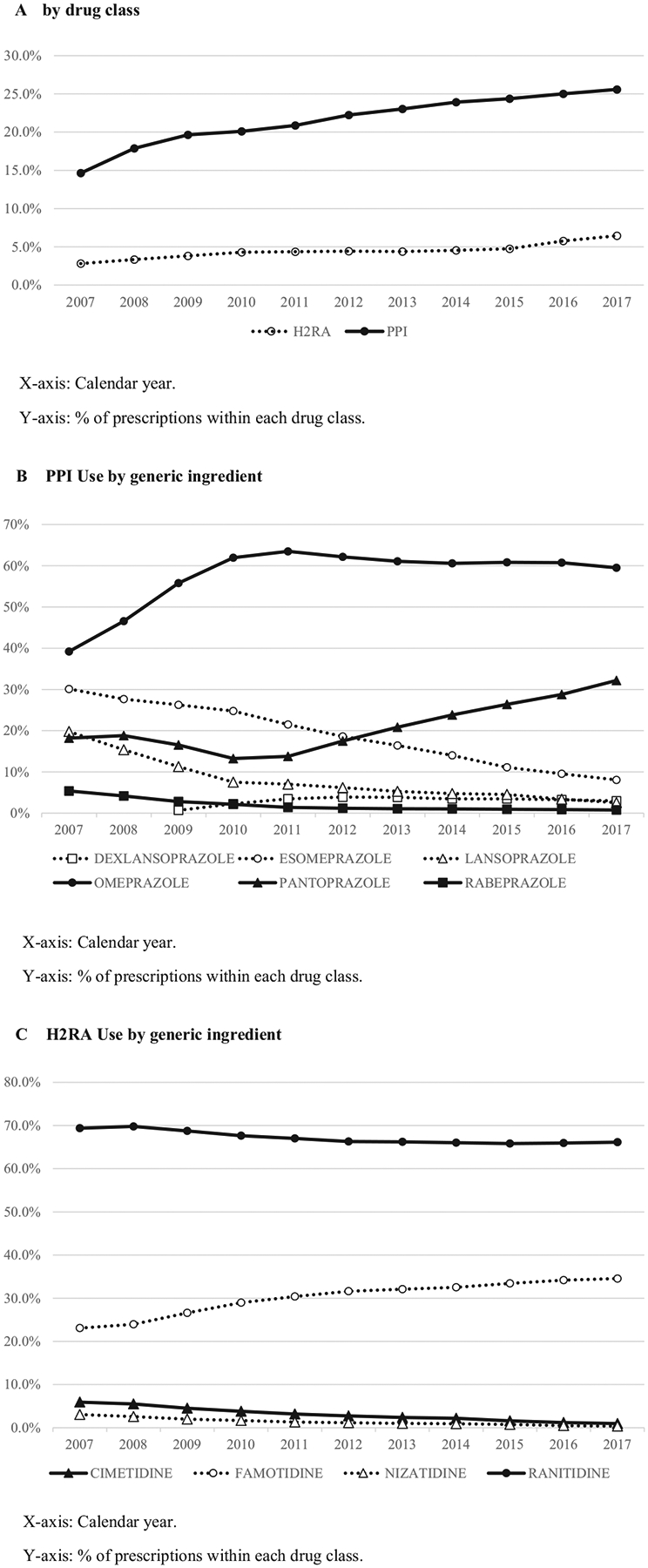
Secular trend of study medication use: (*A*) by drug class; (*B*) proton pump inhibitor (PPI) use by generic ingredient; and (*C*) histamine-2 receptor agonist (H2RA) use by generic ingredient.

During the 11 years of data availability (2007–2017), the proportion of Medicare enrollees taking PPIs grew from 14.6% to 25.6% (Figure 1A). The use of H2RAs increased from 2.8% to 6.4%. These data did not include the use of over-the-counter medications paid with cash, though this is estimated to constitute a small proportion of PPI use (see Supplementary Material C). Omeprazole and ranitidine were most commonly prescribed, accounting for 56.5% and 66.9% of PPIs and H2RAs, respectively. Pantoprazole and famotidine were the second most popular PPI and H2RA, respectively. The use of the other PPIs and H2RAs tended to decline (Figure 1B and C).

Among the 1,930,728 study individuals in the incident cohort, 408,655 (21.2%) had at least 1 claim for a PPI and 131,893 (6.8%) for an H2RA, 72,036 (3.7%) for both, 336,619 (17.4%) for only PPIs, and 59,857 (3.1%) for only H2RAs. Most common prescription duration for PPIs and H2RAs was 30 days, accounting more than 50% of all prescriptions, followed by 90 days (>25%). The time gap between the end of 1 PPI prescription supply and the start of the next, was narrow—a median of 2 days, and mean of 24 days. For H2RAs, the median was also 2, but the mean was 44 days.

Study individuals in the incident cohort were on PPIs and H2RAs for a cumulative median 180 and 90 days, respectively, during a median 1402 days on study (Table 1). Among 2.5 million subjects in the full cohort, twice as many as the incident cohort (840,565) were on PPIs for a median of 424 cumulative days. Thus, the washout removed a disproportionate share of long-term PPI users. The prevalence of chronic diseases and hospitalization in the full cohort was similar to that of the incident cohort, and the prevalence of those chronic diseases was comparable among PPI and H2RA users.

### Primary Analysis

In the Cox regression analysis of the incident cohort, we followed study individuals until they died (4.2%), switched to a capitated plan (14.7%), disenrolled from Medicare (<1%), or reached the end of our study on December 31, 2017 (81.1%). Follow-up lasted a median 3.8 years (8,218,522 patient-years), yielding an overall unadjusted death rate of 9.85 per 1000 person-years. The corresponding mortality rates per 1000 person-years for those who took both PPIs and H2RAs, only PPIs, only H2RAs, and neither PPIs nor H2RAs were 13.41, 14.53, 13.73, and 7.93, respectively.

Table 2 shows the effects of all covariates on all-cause mortality in our Cox regression analysis. Women had a lower mortality risk than men, as expected. Mortality risk appeared to be less in other races than in Whites, but this is likely due to our adjustment for economic status.^26^ Chronic diseases such as cataract, glaucoma, osteoporosis, rheumatoid arthritis or osteoarthritis, asthma, hyperlipidemia, obesity, and hearing impairment were not associated with increased mortality risk. Diseases with worse prognosis (eg, chronic kidney disease; chronic obstructive pulmonary disease; heart failure; breast, colorectal, lung, and endometrial cancers) exhibited a 50% or greater increase in mortality risk. Lung cancer was the worst, with a 4-fold increase in mortality.

We excluded ICU and non-ICU hospital admissions from our primary analyses because they are likely intermediate, rather than confounding variables to the outcome of death. However, in a secondary analysis including hospital and ICU admission as covariates, hospital admissions and ICU admissions almost doubled the risk of death: they had hazard ratios (HRs) of 1.78 (95% CI, 1.72–1.86) and 2.04 (95% CI, 1.98–2.09), respectively. In the full cohort, admissions to the ICU or non-ICU hospital were associated with 5 times the mortality risk of control subjects.

### Effects of Study Medications

Table 3 shows the marginal (ever vs no use) and cumulative (long-term, short-term use vs no use) effects of PPIs and H2RAs on all-cause mortality.

With no lag time, ever use of PPIs showed a small, 10%, increased mortality risk (HR, 1.10; 95% CI, 1.08–1.12). However, with a lag time of 90 days, to correct for protopathic bias, mortality risk of PPI use was close to 0. Furthermore, with a lag time of 90 days, long-term (>6 months cumulative) use of PPIs was protective, reducing the mortality risk by 10% (HR, 0.90; 95% CI, 0.88–0.92). Even without a lag time, the risk reduction with long-term use continued but to a lesser degree at 4% (HR, 0.96; 95% CI, 0.94–0.98). Short-term use (≤6 months cumulative) appeared to increase mortality risk in all of our analyses to 13% (HR, 1.13; 95% CI, 1.10–1.15) with a lag time and to 26% (HR, 1.26; 95% CI, 1.23–1.28) without a lag time in the incident cohorts.

We also examined the full cohort, which had more than twice cumulative days on PPIs. The results were very similar to those in the incident cohort (Table 3). In both the incident and full cohorts, mortality risk of PPI use shifted from negative with long-term cumulative use to positive with short-term use. Short-term users filled their first PPI prescription much closer to their death, at a median 226 (interquartile range, 87–646) days compared with long-term users whose median was 981 (interquartile range, 549–1559) days. A substantial proportion, 23%, of short-term users started their PPI within 90 days of death, whereas no long-term users started it at a short interval. The greater proportionate reduction of risk when lag time was applied to short-term compared with long-term users is consistent with the presence of a protopathic bias.

In the incident cohort analysis, H2RAs, overall, were not associated with mortality risk, with, (HR, 0.91; 95% CI, 0.89–0.94) or without (HR, 0.95, 95% CI, 0.93–0.98), a lag time of 90 days. However, H2RAs are also used in ICU patients and in the full cohort without a lag time, short-term H2RA use exhibited a significant increase in risk (+11%), which also disappeared with the application of 90 days’ lag time (Table 3).

## Discussion

We assessed the effects of PPIs and H2RAs on survival in an incident drug user cohort of nearly 2 million elderly Medicare fee-for-service beneficiaries. We implemented an extended Cox regression analysis to mitigate immortal time bias and a washout to produce an incident cohort to avoid survivor bias. We used a lag time approach to correct for perimortal (protopathic) bias,^27,28^ which occurs when an illness in the latter stages of life influences the exposure to a given drug.^29^

Because PPIs are commonly prescribed for conditions and events that occur in the later stages of life, their effects are especially prone to protopathic bias. About 60% and 40% of nursing home and hospital patients, respectively, are treated with PPIs for stress ulcer prevention^30,31^ and such hospitalizations can presage death. Roughly 30% of dialysis patients take PPIs for peptic symptoms and the prevention of gastrointestinal (GI) bleeding^23^ and dialysis itself carries a 50%, 3-year mortality rate.^32^ Patients taking anticoagulants or antiplatelet medications are another population who receive PPIs for upper GI protection,^24,25^ as do up to 80% of liver cirrhosis patients.^33^ Therefore, conditions with high lethality are associated with receipt of PPIs to prevent upper GI bleeding, which could lead to blame the treatment instead of the underlying condition for the death—a reverse “causality.”^34^

With a lag time approach to protect against that bias, we found no association between PPI use and mortality risk (HR 1.01; 95% CI, 0.99–1.02), and with long-term PPI use (>6 months cumulative) the mortality risk decreased by 10% (HR 0.90; 95% CI, 0.88–0.92). Such risk reduction, changing from negative to null association, is consistent with a protopathic bias. About 23% of short-term users started PPI use within 90 days of their death, and no long-term user did so. Further, for short-term users, the median lag between PPI start and death was over 4 times shorter than that of long-term users.

Our results reinforce those of a recent RCT of PPI that reported no increased mortality in PPI treated patients compared with control subjects^13^ and given our much larger sample size, provide a tighter confidence limits on the conclusion of no harm.

H2RAs exhibited either no effect or improved mortality risk in the incident cohort and were associated with a 10% lower risk when compared with PPIs directly. However, the outcomes of H2RAs use are not the ideal controls for the outcomes of PPI use because of differential usage patterns among patients with high risk conditions. PPIs provide much greater protection against upper GI bleeding risk than H2RAs and are recommended over H2RAs for high-risk ICU prophylaxis.^35^ In one study, patients given H2RAs were younger and had lower Charlson comorbidity index than those given PPIs.^36^

The statistical analyses used in the VA studies^10,11^ were excellent and without flaws. However, the databases may have had limitations.

First, based on age range alone, at least half of their subjects were Medicare eligible during the observation period. Such patients obtain 47% of their care outside of the VA system differentially by medical problem.^14^ These Veterans with 1 hospitalization then obtain 82% of follow-up care outside the VA,^37^ and these data are not available, introducing potential ascertainment bias.

Second, the VA’s study results may not be generalizable to non–VA care patients. The literature suggests that patients in the VA care system have higher comorbidity and are 14 times more likely to have poorer health statuses than the general population.^38^ In the VA study,^10^ the death rate of 44.7 per 1000 patient-years and death prevalence of 23% were much higher than the death rate (18.7 per 1000 patient-years) and prevalence (9%) in our special cohort of male patients. The excess mortality based on difference in mortality rates between patients taking only PPIs and those taking only H2RAs was 45.2 per 1000 patient-years in the VA study^11^ and only 0.9 per 1000 patient-years in our study.

Our study had several limitations, including the potential for residual confounding. We lacked direct test measures to validate diagnostics codes and depended completely on billing diagnoses, which can distort analyses. Our results differed from those of the VA but heterogeneity in populations, analytic design, length of follow-up, and set of covariates between 2 studies can well produce opposite conclusions.^39^

Given the usage patterns of PPIs in patients with conditions and admissions that may presage death, correction of protopathic bias with a lag time approach is important and observational studies should use it more often. Our results aligned well with the only RCT of PPIs and provide confidence limits around the conclusion of no harm closer to zero than theirs.^13^ Our study adds to the recent RCT evidence and other reports that PPIs are not associated with increased mortality risk.^40^

## Supplementary Material

Supplementary Material

Supplementary Material

Note: To access the supplementary material accompanying this article, please click here.

## Figures and Tables

**Table 1.** Outcome and Patient Characteristics by Use of Study Medications

	Overall (N = 1,930,728)	Both (n = 72,036)	PPI only (n = 336,619)	H2RA only (n = 59,857)	Neither (n = 1,462,216)
Death	80,972 (4.2)	5789 (8.0)	25,774 (7.7)	4063 (6.8)	45,346 (3.1)
Censored at HMO entry	284,392 (14.7)	6463 (9.0)	36,739 (10.9)	6961 (11.6)	234,229 (16.0)
Censored at disenrollment	230 (0.0)	6 (0.0)	24 (0.0)	3 (0.0)	197 (0.0)
Censored at December 31, 2017	1,565,134 (81.1)	59,778 (83.0)	274,082 (81.4)	48,830 (81.6)	1,182,444 (80.9)
Years of follow-up, median (total)	3.8 (8,218,552)	5.8 (431,677)	4.9 (1,773,945)	4.5 (295,880)	3.5 (5,717,050)
Deaths per 1000 person-years	9.85	13.41	14.53	13.73	7.93
Days of follow-up	1402.0 (823.0–2101.0)	2132.0 (1492.0–2954.0)	1795.0 (1218.0–2649.0)	1645.0 (1065.0–2467.0)	1280.0 (731.0–1954.5)
Days on PPI	180.0 (60.0–612.0)	268.0 (90.0–757.0)	178.0 (60.0–579.0)	N/A	N/A
Days on H2RA	90.0 (46.0–304.0)	120.0 (60.0–344.0)	N/A	72.0 (30.0–259.0)	N/A
ICU ever	271,350 (14.1)	21,500 (29.8)	83,392 (24.8)	14,884 (24.9)	151,574 (10.4)
Hospital ever	612,171 (31.7)	40,432 (56.1)	167,622 (49.8)	28,062 (46.9)	376,055 (25.7)
Female	1,112,530 (57.6)	48,664 (67.6)	205,441 (61.0)	37,134 (62.0)	821,291 (56.2)
White	1,582,990 (82.0)	56,117 (77.9)	276,468 (82.1)	47,011 (78.5)	1,203,394 (82.3)
Black	131,392 (6.8)	5686 (7.9)	21,819 (6.5)	5443 (9.1)	98,444 (6.7)
Hispanic	96,733 (5.0)	5680 (7.9)	19,003 (5.6)	3803 (6.4)	68,247 (4.7)
Asian	48,415 (2.5)	2755 (3.8)	9779 (2.9)	1797 (3.0)	34,084 (2.3)
Other	71,198 (3.7)	1798 (2.5)	9550 (2.8)	1803 (3.0)	58,047 (4.0)
Ever dual	263,863 (13.7)	17,630 (24.5)	55,791 (16.6)	12,409 (20.7)	178,033 (12.2)
Nondual LIS	52,844 (2.7)	2164 (3.0)	8808 (2.6)	1778 (3.0)	40,094 (2.7)
Nondual no LIS	1,614,021 (83.6)	52,242 (72.5)	272,020 (80.8)	45,670 (76.3)	1,244,089 (85.1)
Living in rural area	432,165 (22.4)	18,179 (25.2)	80,033 (23.8)	14,936 (25.0)	319,017 (21.8)
AMI	44,933 (2.3)	4085 (5.7)	13,870 (4.1)	2846 (4.8)	24,132 (1.7)
Atrial fibrillation	156,051 (8.1)	9937 (13.8)	42,821 (12.7)	6816 (11.4)	96,477 (6.6)
Cataract	982,757 (50.9)	47,895 (66.5)	204,075 (60.6)	34,206 (57.1)	696,581 (47.6)
Chronic kidney disease	397,173 (20.6)	25,844 (35.9)	102,792 (30.5)	18,065 (30.2)	250,472 (17.1)
COPD	285,913 (14.8)	22,437 (31.1)	82,398 (24.5)	13,701 (22.9)	167,377 (11.4)
Heart failure	216,895 (11.2)	17,759 (24.7)	64,982 (19.3)	11,320 (18.9)	122,834 (8.4)
Diabetes	553,577 (28.7)	29,062 (40.3)	121,829 (36.2)	21,839 (36.5)	380,847 (26.0)
Glaucoma	312,575 (16.2)	15,204 (21.1)	62,764 (18.6)	10,644 (17.8)	223,963 (15.3)
Hip/pelvic fracture	18,296 (0.9)	1630 (2.3)	5546 (1.6)	999 (1.7)	10,121 (0.7)
Ischemic heart disease	541,229 (28.0)	35,991 (50.0)	141,668 (42.1)	23,978 (40.1)	339,592 (23.2)
Depression	429,246 (22.2)	29,974 (41.6)	114,034 (33.9)	18,660 (31.2)	266,578 (18.2)
Alzheimer’s disease or dementia	83,228 (4.3)	7705 (10.7)	24,324 (7.2)	4617 (7.7)	46,582 (3.2)
Osteoporosis	229,513 (11.9)	15,973 (22.2)	55,549 (16.5)	9312 (15.6)	148,679 (10.2)
Rheumatoid Arthritis/osteoarthritis	797,821 (41.3)	47,572 (66.0)	194,784 (57.9)	32,047 (53.5)	523,418 (35.8)
Stroke/transient ischemic attack	120,982 (6.3)	9,802 (13.6)	33,476 (9.9)	6419 (10.7)	71,285 (4.9)
Breast cancer	96,178 (5.0)	5025 (7.0)	20,818 (6.2)	3584 (6.0)	66,751 (4.6)
Colorectal cancer	31,985 (1.7)	1979 (2.7)	8799 (2.6)	1327 (2.2)	19,880 (1.4)
Prostate cancer	79,048 (4.1)	2975 (4.1)	15,335 (4.6)	2541 (4.2)	58,197 (4.0)
Lung cancer	29,767 (1.5)	2397 (3.3)	9989 (3.0)	1562 (2.6)	15,819 (1.1)
Endometrial cancer	16,006 (0.8)	845 (1.2)	3727 (1.1)	696 (1.2)	10,738 (0.7)
Anemia	632,512 (32.8)	42,038 (58.4)	171,804 (51.0)	27,460 (45.9)	391,210 (26.8)
Asthma	169,066 (8.8)	14,166 (19.7)	48,239 (14.3)	7934 (13.3)	98,727 (6.8)
Hyperlipidemia	1,357,973 (70.3)	61,404 (85.2)	274,990 (81.7)	47,635 (79.6)	973,944 (66.6)
Hyperplasia	263,770 (13.7)	11,773 (16.3)	57,408 (17.1)	9266 (15.5)	185,323 (12.7)
Hypertension	1,315,427 (68.1)	60,643 (84.2)	271,783 (80.7)	47,798 (79.9)	935,203 (64.0)
Hypothyroidism	404,760 (21.0)	22,457 (31.2)	90,409 (26.9)	15,261 (25.5)	276,633 (18.9)
Alcohol use disorders	58,163 (3.0)	3708 (5.1)	16,938 (5.0)	2656 (4.4)	34,861 (2.4)
Anxiety disorders	324,594 (16.8)	25,504 (35.4)	90,317 (26.8)	14,928 (24.9)	193,845 (13.3)
Bipolar disorder	35,546 (1.8)	2962 (4.1)	9768 (2.9)	1774 (3.0)	21,042 (1.4)
Major depressive disorder	314,281 (16.3)	23,824 (33.1)	87,850 (26.1)	14,200 (23.7)	188,407 (12.9)
Drug use disorder	41,692 (2.2)	3751 (5.2)	12,763 (3.8)	2175 (3.6)	23,003 (1.6)
Personality disorders	20,191 (1.0)	1604 (2.2)	5343 (1.6)	918 (1.5)	12,326 (0.8)
Schizophrenia	29,916 (1.5)	2955 (4.1)	8738 (2.6)	1782 (3.0)	16,441 (1.1)
Epilepsy	31,398 (1.6)	2540 (3.5)	8401 (2.5)	1825 (3.0)	18,632 (1.3)
Cystic fibrosis	23,583 (1.2)	1657 (2.3)	6185 (1.8)	998 (1.7)	14,743 (1.0)
Fibromyalgia, chronic pain	410,617 (21.3)	29,798 (41.4)	110,801 (32.9)	17,535 (29.3)	252,483 (17.3)
Viral hepatitis (general)	22,840 (1.2)	1584 (2.2)	6129 (1.8)	1069 (1.8)	14,058 (1.0)
Liver disease cirrhosis	131,378 (6.8)	11,465 (15.9)	43,668 (13.0)	5787 (9.7)	70,458 (4.8)
Leukemias and lymphomas	32,244 (1.7)	2142 (3.0)	8854 (2.6)	1443 (2.4)	19,805 (1.4)
Migraine	72,304 (3.7)	6022 (8.4)	20,359 (6.0)	3014 (5.0)	42,909 (2.9)
Mobility impairments	40,989 (2.1)	3713 (5.2)	12,044 (3.6)	2812 (4.7)	22,420 (1.5)
Obesity	429,848 (22.3)	23,340 (32.4)	101,087 (30.0)	17,493 (29.2)	287,928 (19.7)
Opioid use disorder	24,927 (1.3)	2556 (3.5)	8277 (2.5)	1311 (2.2)	12,783 (0.9)
Peripheral vascular disease	196,669 (10.2)	16,101 (22.4)	55,318 (16.4)	9931 (16.6)	115,319 (7.9)
Tobacco use disorders	216,083 (11.2)	12,531 (17.4)	51,439 (15.3)	9527 (15.9)	142,586 (9.8)
Pressure ulcers	67,398 (3.5)	5610 (7.8)	19,980 (5.9)	3778 (6.3)	38,030 (2.6)
Deafness	140,182 (7.3)	9424 (13.1)	35,156 (10.4)	5661 (9.5)	89,941 (6.2)

NOTE. Values are n (%) or median (interquartile range), unless otherwise indicated.

AMI, acute myocardial infarction; COPD, chronic obstructive pulmonary disease; H2RA, histamine-2 receptor agonist; HMO, Health Maintenance Organization; ICU, intensive care unit; LIS, low-income-subsidy; N/A, Not applicable; PPI, proton pump inhibitor.

**Table 2.** HRs of All-Cause Mortality for Each Covariate

Covariate	Reference	HR (95% CI)
Age at part D entry		1.04 (1.03–1.05)^a^
Female	Male	0.60 (0.59–0.61)^b^
Black	White	1.00 (0.98–1.03)
Hispanic		0.69 (0.67–0.72)^b^
Asian		0.62 (0.58–0.65)^b^
Other		0.94 (0.90–0.98)^c^
Dual ever	Nondual no LIS	1.40 (1.37–1.43)^a^
Nondual LIS		1.48 (1.44–1.54)^a^
Living in rural area	No	1.02 (1.01 −1.04)^d^
AMI	No	0.99 (0.95–1.02)
Atrial fibrillation	No	1.19 (1.17–1.22)^a^
Cataract	No	0.81 (0.80–0.82)^b^
Chronic kidney disease	No	1.48 (1.46–1.51)^a^
COPD	No	1.52 (1.49–1.55)^a^
Heart failure	No	1.53 (1.50–1.56)^a^
Diabetes	No	1.18 (1.16–1.20)^a^
Glaucoma	No	0.86 (0.84–0.88)^b^
Hip/pelvic fracture	No	1.38 (1.32–1.44)^a^
Ischemic heart disease	No	0.97 (0.96–0.99)^c^
Depression	No	1.02 (1.00–1.04)
Alzheimer’s disease or dementia	No	1.97 (1.92–2.02)^a^
Osteoporosis	No	0.93 (0.91–0.95)^b^
Rheumatoid arthritis/osteoarthritis	No	0.65 (0.64–0.67)^b^
Stroke/transient ischemic attack	No	1.07 (1.04–1.10)^a^
Breast cancer	No	1.64 (1.59–1.69)^a^
Colorectal cancer	No	1.81 (1.75–1.87)^a^
Prostate cancer	No	1.19 (1.15–1.23)^a^
Lung cancer	No	4.40 (4.29–4.52)^a^
Endometrial cancer	No	2.47 (2.35–2.61)^a^
Anemia	No	1.54 (1.52–1.57)^a^
Asthma	No	0.76 (0.74–0.78)^b^
Hyperlipidemia	No	0.59 (0.58–0.60)^b^
Hypertension	No	1.11 (1.09–1.13)^a^
Hyperplasia	No	0.75 (0.73–0.76)^b^
Hypothyroidism	No	0.91 (0.89–0.92)^b^
Alcohol use disorders	No	1.02 (1.00–1.05)
Anxiety disorders	No	0.99 (0.97–1.01)
Bipolar disorder	No	0.92 (0.88–0.95)^b^
Drug use disorder	No	0.91 (0.87–0.94)^b^
Personality disorders	No	1.03 (0.96–1.10)
Schizophrenia	No	1.21 (1.17–1.26) ↟
Epilepsy	No	1.17 (1.12–1.21) ↟
Cystic fibrosis	No	1.06 (1.00–1.12)
Fibromyalgia	No	0.91 (0.89–0.93)^b^
Viral hepatitis (general)	No	1.02 (0.98–1.07)
Liver disease cirrhosis	No	1.22 (1.19–1.25)^a^
Leukemias and lymphomas	No	1.86 (1.80–1.93)^a^
Migraine	No	0.61 (0.58–0.64)^b^
Mobility impairments	No	1.26 (1.22–1.31)^a^
Obesity	No	0.92 (0.90–0.93)^b^
Opioid use disorder	No	0.99 (0.94–1.04)
Peripheral vascular disease	No	1.09 (1.07–1.11)^a^
Tobacco use disorders	No	1.47 (1.44–1.50)^a^
Pressure ulcers	No	1.66 (1.62–1.70)^a^
Deafness	No	0.78 (0.76–0.81)^b^

AMI, acute myocardial infarction; CI, confidence interval; COPD, chronic obstructive pulmonary disease; HR, hazard ratio; LIS, low-income-subsidy.

a Very significantly high with *P* value <.001.

b Very significantly low with *P* value <.001.

c Significantly low with .001 ≤ *P* value < .05.

d Significantly high with .001 ≤ *P* value < .05.

**Table 3.** HR of All-Cause Mortality for Each Study Medication by Duration of Drug Exposure

		Incident cohort		Full cohort	
		No lag time	Lag time of 90 d	No lag time	Lag time of 90 d
Covariate	Reference	HR (95% CI)	HR (95% CI)	HR (95% CI)	HR (95% CI)
**Ever on PPI**	Never on PPI	1.10 (1.08–1.12)^a^	1.01 (0.99–1.02)	1.10 (1.08–1.11)^a^	0.98 (0.97–0.99)^b^
**Short-term PPI use**	Never on PPI	1.26 (1.23–1.28)^a^	1.13 (1.10–1.15)^a^	1.29 (1.27–1.31)^a^	1.10 (1.08–1.11)^a^
**Long-term PPI use**		0.96 (0.94–0.98)^b^	0.90 (0.88–0.92)^c^	0.94 (0.92–0.95)^c^	0.87 (0.86–0.89)^c^
**Ever on H2RA**	Never on H2RA	0.95 (0.93–0.98)^c^	0.91 (0.89–0.94)^c^	1.02 (1.01–1.04)^d^	0.97 (0.95–0.99)^b^
**Short-term H2RA use**	Never on H2RA	1.03 (0.99–1.06)	0.98 (0.94–1.01)	1.11 (1.09–1.14)^a^	1.02 (1.00–1.04)
**Long-term H2RA use**		0.88 (0.85–0.91)^c^	0.86 (0.82–0.89)^c^	0.94 (0.92–0.96)^a^	0.92 (0.90–0.94)^c^
**Ever on PPI**	Ever on H2RA	1.16 (1.12–1.20)^a^	1.10 (1.06–1.14)^a^	1.07 (1.05–1.10)^a^	1.01 (0.99–1.03)

NOTE. For incident cohort analyses, short-term/long-term PPI use: ≤6/>6 months cumulative use; short-term/long-term H2RA use: ≤4/>4 months cumulative use; for full cohort analyses: short-term/long-term PPI use: ≤1/>1 year cumulative use; short-term/long-term H2RA use: ≤6/>6 months cumulative use for incident cohort.

CI, confidence interval; H2RA, histamine-2 receptor agonist; HR, hazard ratio; PPI, proton pump inhibitor.

a Very significantly high with *P* value <.001.

b Significantly low with .001 ≤ *P* value < .05.

c Very significantly low with *P* value <.001.

d Significantly high with .001 ≤ *P* value < .05.

## What You Need to Know

### Background

Observational studies linked proton pump inhibitors with increased risk of mortality may attribute death to the treatment rather than the conditions that are treated with PPIs, perimortal (protopathic) bias.

### Findings

After controlling for protopathic bias which occurs when an intervention is associated with prodromal signs of that outcome, PPI did not exhibit the increased risk of death.

### Implications for patient care

Given the usage patterns of PPIs in patients with conditions that may presage death, PPI use corrected for protopathic bias did not exhibit increased risk of death.

## Abbreviations used in this paper:

CI: confidence interval
FPL: Federal Poverty Line
GI: gastrointestinal
H2RA: histamine-2 receptor agonist
ICU: intensive care unit
HR: hazard ratio
LIS: low-income-subsidy
PPI: proton pump inhibitor
VA: Department of Veterans Affairs
