# Supplementary material for: The Mortality Risk of Proton Pump Inhibitors in 1.9 Million US Seniors: An Extended Cox Survival Analysis
Source: Clin Gastroenterol Hepatol. Author manuscript; Available in PMC 2025 Aug 27. (PMC12381940; doi:10.1016/j.cgh.2021.01.014)
Supplement: Supplementary Material [file NIHMS2105034-supplement-Supplementary_Material.docx]

**Supplementary Material**

1. *Special cohort*

We started all subjects at or close to age of 65 over the 11 years of our study. Data availability started in 2007 and ended in 2017, so follow-up time became progressively shorter with each successive entry year. We created a subset only including males who entered in 2007 and 2008 (10-11 years of follow up) to obtain a population whose death rate would be more comparable to that of the VA study cohort, whose subjects were 94% males, started at mean (standard deviation) ages (61±15), and were followed for 7 years.^1^ For this subset, we obtained the prevalence of death, unadjusted death rate to compare with those in the VA Study. In this cohort, the prevalence of death, (11.2%), the mortality rates (14.5 per 1000 person-years) and the comparable figure for those who took both (17.76 per 1000 person-years), only PPIs (18.10 per 1000 person-years), only H2Ras (17.23 per 1000 person-years) and none (12.23 per 1000 person-years) were greater than in our primary cohorts because of the exclusions. Importantly, the excess mortality per 1000 person-years taking only PPIs (vs. only H2RAs) from this cohort was miniscule (0.87 deaths/1000 person-years) compared to 45.20 excessive deaths/1000 person-years in the VA study.

1. *Time-varying propensity scores*

In order to mitigate treatment bias toward the use of either of the study drug classes, we used multiple logistic regressions to develop *time-varying* propensity scores (PSs) for each drug class as a function of demographics, socio-economics and presence of chronic condtions.^2^ We iteratively estimated the PSs every 6 months among the patients who remain in follow-up considering time-fixed demographics and time-varying socio-economics and chronic conditions that preceded the end of a given 6-month cycle.^3^

1. *Over-the-Counter Medication data*

During our observation period, individuals could purchase PPIs and H2RAs over-the-counter (OTC) without prescriptions. In general, the CMS has no access to such “cash” purchases. However, from the beginning of Part D (October 2006), with a physician’s prescription, 100% of Medicare fiscal intermediaries paid the for most (omeprazole, esomeprazole, lansoprazole) OTC PPIs, and a smaller percent of intermediaries paid for OTC H2RAs.^4^ So we assume Medicare’ data regarding PPI use is reasonably complete, and that of H2RA’s less so. In an attempt to verify our assumption about the PPIs, we obtained summary data from a large pharmacy chain about the proportion of OTC PPI pills paid out of pocket versus bought with a prescription and paid by Medicare for the full year ending October 2017. About 86% of all PPI pills sold in a large national pharmacy chain, were prescription rather than OTC in the year ending October 31, 2017. For dual eligible enrollees, Medicare pays almost in full for both PPIs and H2RAs so we also compared the usage data for both PPIs and H2RAs between Medicare-Medicaid (dual) eligible vs regular Medicare beneficiaries. Among those who took either PPIs or H2RAs, the difference in prescriptions of PPIs (78.6%) and H2RAs (21.4%) among dual-eligible enrollees was smaller than the difference among non-dual enrollees (87.7% and 12.3% respectively), likely due to the better coverage of OTC H2RAs among the latter. We also report the descriptive statistics for supply durations, and time between the end of prescription duration and the start of the next prescription as well as the proportion of patients who were on both H2RAs at one time and PPIs at another.

**References**

1. Xie Y, Bowe B, Li T, et al. Risk of death among users of Proton Pump Inhibitors: a longitudinal observational cohort study of United States veterans. BMJ Open 2017;7:e015735.

2. Rosenbaum PR, Rubin DB. The Central Role of the Propensity Score in Observational Studies for Causal Effects. Biometrika 1983;70:41–55.

3. Wyss R, Gagne JJ, Zhao Y, et al. Use of Time-Dependent Propensity Scores to Adjust Hazard Ratio Estimates in Cohort Studies with Differential Depletion of Susceptibles. Epidemiology 2020;31:82–89. Available at: http://journals.lww.com/00001648-202001000-00009 [Accessed July 17, 2020].

4. Piper K. Over-the-Counter Drugs in Medicare Part D: Impact of OTCs on Medicare Drug Plans, Medicaid Programs, Pharmacies, and Drug Manufacturers : Piper Report. Available at: https://piperreport.com/blog/2006/06/03/overthecounter_1/ [Accessed November 1, 2019].
